# Humidity Induces Changes in the Dimensions of Hydrogel-Coated Wool Yarns

**DOI:** 10.3390/polym10030260

**Published:** 2018-03-02

**Authors:** Lanlan Wang, Artur Cavaco-Paulo, Bo Xu, Madalena Martins

**Affiliations:** 1 International Joint Research Laboratory for Textile and Fiber Bioprocesses, Jiangnan University, Wuxi 214122, China; 15852776701@163.com (L.W.); artur@deb.uminho.pt (A.C.-P.); 2 Centre of Biological Engineering (CEB), University of Minho, Campus of Gualtar, 4710-057 Braga, Portugal; 3 Key Laboratory of Eco-Textiles, Ministry of Education, Jiangnan University, Wuxi 214122, China

**Keywords:** hydrogel, wool yarns, photocrosslinking, UV polymerization

## Abstract

Polymeric hydrogel based on acrylic acid (AA) and *N*,*N*-dimethylacrylamide (DMAA) was prepared by photopolymerization reaction, using nano-alumina as the inorganic crosslinker. Hydrogel-coated wool yarns determine their dimensional changes under humidity conditions. Surface morphology of the hydrogel-coated wool yarns was carried out using SEM microscopy. The hydrogel was further characterized by Fourier transformer infrared spectrum (FTIR), gel permeation chromatography (GPC), differential scanning calorimetry (DSC), thermogravimetry (TG) and differential thermogravimetry (DTG). This contribution showed that UV-initiated polymerization coating wool yarns can change the functional properties of wool fibers.

## 1. Introduction

Keratin fiber is a biocompatible and huge network polymer material. Its structure consists of a cuticle, cortex and cell membrane complex [1]. This protein composes the soft keratins as a structural component of skin (e.g., from epidermis), and the hard keratins as a major component of hair, epidermis, wool, horns and also feathers, claws, and beaks of birds and reptiles [2]. Various studies related to wool keratin structure have been extended to studies related to human hair keratin since its microstructure is analogous to wool keratin. Keratin fibers from mammals are extensively crosslinked structures with disulfide bonds, which is an important parameter for their cosmetic point of view. Hair and wool fibers are comprised of the alpha-keratin which is a fibrous and helical protein, having a coherent structure by polypeptide chains composed of a higher amount of sulfur atoms [3]. The molecules in the polypeptide chain are joined together between the carboxyl group of one molecule and the amine group of the other, resulting in a functional group CONH as the peptide link [4].

Hydrogels with unique and versatile properties have been applied in a vast number of potential applications including biomedical, cosmetic, pharmaceutical, and environmental fields. Hydrogel is a three-dimensional hydrophilic polymer with the capacity to swell and hold large amounts of water or biological fluids [5]. Its three-dimensional network is formed by crosslinking polymer chains. Crosslinking can be provided by covalent bonds, hydrogen bonding, Vander Waals interactions or physical entanglements. The type of crosslinking method can be chemical, physical or ionic [6], depending on the desired application of the hydrogel [7].

Hydrogels have unique properties of biocompatibility, elastic properties and high water content, and the ability to tailor and control these properties. Their physical properties are similar to natural living tissue, exhibiting flexibility, high water content, porosity, and soft consistency. Their inherent biocompatibility and capacity for swelling makes them suitable for a wide range of applications [8]. Some of the most dynamic applications are in drug delivery systems [9], wound dressings [8], tissue engineering [10], thickening agents for cosmetics [11], in water absorbency and water retention [12], and manufacture of personal care such as hygienic products [13]. Hydrogels have also been applied as intelligent, controlling release or being sensitive to different stimuli conditions, such as temperature, pH, enzymatic activities, light and electric fields [11,14]. Based on hydrogel preparation methods, they can be classified as homopolymer gels or copolymer gels, and can be prepared from synthetic or/and natural polymers [15]. Hydrogels as three-dimensional crosslinked hydrophilic polymer networks have a capacity for swelling or de-swelling reversibly in water and retaining a large volume of liquid in their swollen state [13,16]. Photopolymerization of hydrogels is of particular interest in biomedical applications because of its in situ and non-toxic gelation [15]. Light-activated polymerization is typically carried out with an appropriate wavelength, whereby free radicals attack vinyl groups on precursor macromolecules, leading to the formation of covalent bonds that crosslink the hydrogel network under UV light exposure [15,17,18].

Active hair care research has been carried out based on wool science, developing attractive solutions such as hair color, permanent waving, and straightening products [19]. There is an ongoing need to develop new products for hair care cosmetic use, such as changing its morphology characteristics. In this context, the aim of this study was the development of a hydrogel formulation to coat the surface of wool yarn and test its capacity for stretching and curling induced by humidity. From a cosmetic point of view, the hydrogel coating on wool yarn could have high potential interest in hair care use since it explores dimensional changes of the hair fiber under humidity conditions.

## 2. Materials and Methods

### 2.1. Materials

Merino wool yarns with average diameter of 36 mm were obtained from Wuxi Lida Spinnery, Wuxi, Jiangsu, China, The urea and sodium hydroxide were obtained by Sinopharm, China chemical reagent. Nano-alumina with size ranges from 5–10 nm and nano-titania with size ranges from 10–30 nm were purchased by Hangzhou Wanjing, Hangzhou, China, New Materials. Acrylamide (AM) and acrylic acid (AA) were provided by Aladdin Industrial Corporation, Shanghai, China. Bis-Acrylamide (BIS) was supplied by Shanghai Weita, Jiangsu, China. *N*,*N*-dimethylacrylamide (DMAA) and *N*-isopropylacrylamide (NIPAM) were obtained by TCI, Shanghai, China. Irgacure 2959 [2-hydroxy-4-(2-hydroxyethoxy)-2-methylpropiophenone] was provided by Energy Chemical, Tehran, Iran. All the other reagents were used as received, without further purification and were obtained from Sigma-Aldrich, St. Louis, MI, USA.

### 2.2. Hydrogel Formulations and Coating on Wool Yarn

The wool yarn samples were pre-treated for further coating by the hydrogel formulations. Firstly, the wool yarn was immersed in four different solutions with urea and sodium hydroxide at pH 8–10 for two hours. The wool yarn samples were pre-treated with 8 M urea, 8 M urea with 0.0025 M NaOH, 8 M urea with 0.005 NaOH, 8 M urea with 0.025 NaOH and 8 M urea with 0.05 NaOH which were coded as a, b, c, d, and e, respectively.

**Figure 1:**
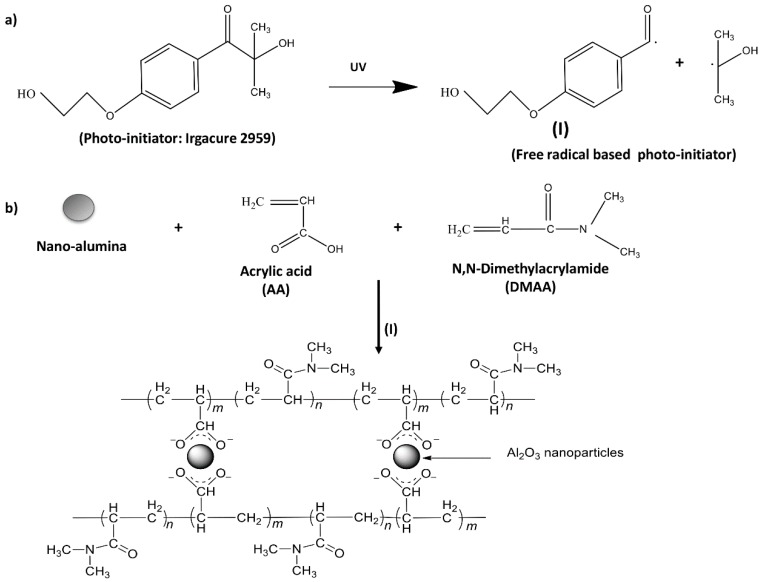
(**a**) Formation of the initiator radical; (**b**) Formation of the hydrogel with nano-alumina particles.

Hydrogel formulations were based on the monomers (*N*,*N*-dimethylacrylamide and acrylic acid), crosslinking agents (bis-acrylamide, nano-alumina oxide and nano-titania oxide) and photo-initiator which were dissolved in distilled water. The reaction solution was further stirred for about 30 min until completely dissolved under nitrogen atmosphere. Then, the reaction mixture was exposed to a long-wavelength UV lamp in a chamber (Scientz03-II, Xinzhi Corporation, Taizhou, China) at 365 nm (8 W/cm^2^) for 30 min. Figure 1 represents the reaction mechanism to form the hydrogel.

The resultant hydrogels were stacked in a refrigerator at 4 °C before further characterization.

### 2.3. Morphological Characterization of the Hydrogel-Coated Wool Yarn

The morphological characterization of hydrogel-coated wool yarns was conducted using scanning electron microscopy (SEM) technique in an electron microscope model SU1510 (Japan Hitachi Co. Ltd., Tokyo, Japan) at 2000× and 500× magnification.

### 2.4. Characterization of Hydrogel Synthesized by Photopolymerization

Gel permeation chromatography (GPC) was used to determine the molecular weight and molecular weight distribution of the polymer. GPC analysis was carried out with columns TSK GMPWxl using Shimadzu RID-20A equipment (Shimadzu Corporation of Japan, Kyoto, Japan). The hydrogel was immersed in ethanol solution and placed it for 24 h. The mobile phase consisted of a solution containing sodium nitrate (0.05 mg/L) and sodium azide (0.2%). Calibration was carried out with narrow molecular weight distribution of polyethylene oxide standards (ranging from 106 Da to 100,000 Da).

Fourier transforms infrared (FT-IR) analysis was used for spectral characterization of the synthesized hydrogel. The absorption spectra in the infrared region was recorded using KBr pellets on a Fourier transform infrared spectrophotometer (Nicolet is10, Thermo Fisher Scientific, Waltham, MA, USA). The spectrum was recorded in the range of 4000 cm^−1^–500 cm^−1^.

### 2.5. Thermal Analysis: Differential Scanning Calorimetry (DSC) and Thermogravimetry Analysis (TGA)

The hydrogel, in wet and dry state, was analyzed by DSC and TGA, using an equipment of Q200 V24.8 Build 120 and Q500 V20.13 Build 39, respectively. Thermal analysis was carried out using samples weighted 5 ± 0.5 mg ranging in temperature from 25 °C to 400 °C for DSC and from 40 to 800 °C for TGA, at a heating rate of 10 °C/min in dynamic nitrogen atmosphere.

### 2.6. Mechanical Properties Evaluation: Tensile Strength and Elongation of Hydrogel-Coated Wool Yarn

Mechanical tests of the hydrogel-coated wool yarns samples were evaluated following guidelines outlined in ASTM D1445-95 for fiber tensile and elongation tests. The measurements were performed in an Instron 4505 tensile tester, with a 2.5 N load cell. For each type of wool yarn sample, fifteen single wool yarns were individually mounted in the tensile jig, using a paper device with a fixed gauge length of 50 mm. All the samples were kept under the same conditions before the measurements. The measurements were performed with a constant rate of 1.5 mm min^−1^ until breakage. The measurements were performed assuming an average mean fiber diameter of 20 µm, value that was obtained through previous measurements with light microscopy. The data recorded in the equipment (applied load against extension) was converted to stress (load/unit area) against strain (% extension). Percentage of elongation was calculated by the elongation at the moment of rupture divided by the initial length measurement and then multiplied by 100.

## 3. Results and Discussion

**Figure 2:**
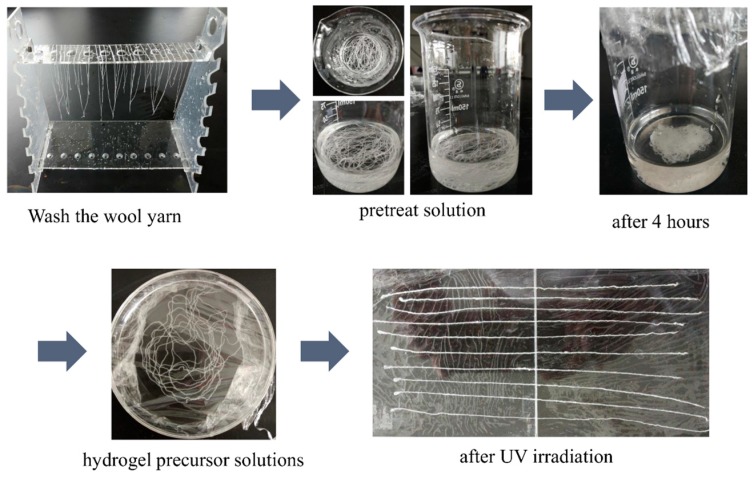
The procedure of wool keratin yarns coated by hydrogel precursor: from preparation of wool yarns to polymerization reaction (UV irradiation).

The photo-crosslinking methodology by UV light irradiation has been widely used in several applications, including numerous types of coatings as well as in biomedical applications. Firstly, the wool yarns were washed thoroughly using detergent (Whitecat detergent, Shanghai Hutchison Whitecat Co. Ltd., Shanghai, China) and then were coated on hydrogel formulation by polymerization reaction Figure 2. The polymerization was conducted by UV irradiation (365 nm). The use of photo-initiators determines the initiation of the polymerization mechanism since their excitation under the UV irradiation leads to the formation of the radicals to initiate the mechanism [20].

The wool yarns were coated by different hydrogel formulations and were evaluated under length, elongation, and strength variation (Table 1). The results obtained for each hydrogel-coated wool yarn were analyzed after polymerization, dried and at 95% humidity state, respectively. The quantity of crosslinker and monomer in the hydrogel formulation was previously optimized (data not shown). It was used different cross-linkers and monomers in the hydrogel formulation, alumina with acrylic acid and titania with acrylamide (formulation 1 and 2, respectively). It was found that the wool yarns coated with the formulation 1 presents a slight improvement in strength and elongation properties when compared with untreated yarn, at 95% humidity condition. Otherwise the yarns coated with formulation 2 presented slight improvement in strength after polymerization condition. From these results it was found that further optimization of the hydrogel formulation as well as a pre-treatment to enhance the swelling of wool yarns, it is required for the variation of the dimensions of the yarns.

The pre-treatment of wool yarns was established to improve the swelling properties and evaluate the length variation of hydrogel-coated wool. To that, the wool yarns were washed and followed by urea treatment (8 mol/L urea solution followed by shaking at 50 °C for 1 h). Then, the yarns were immersed on hydrogel precursor formulations: cross-linker plus monomer and 0.1% of the photo-initiator, during 1 h in the dark environment. After immersion, yarns were exposed to UV irradiation at 365 nm to trigger the free radical polymerization. The obtained yarns were dried at 50 °C, were wetted at four different humidity conditions: 50%, 70%, 90% and 100% humidity (Table 2A) and were subjected to wetting-drying cycles (Table 2B). Hydrogel precursor formulations: BIS-AM (0.4% BIS + 1 M AM+); BIS-NIPAM (0.4% BIS + 1 M NIPAM); Nano-TiO_2_-AM (15% TiO_2_ + 0.5 M AM) and Nano-Al_2_O_3_–AD (10% Al_2_O_3_ + 1% AA + 9% DMAA).

**Figure 3:**
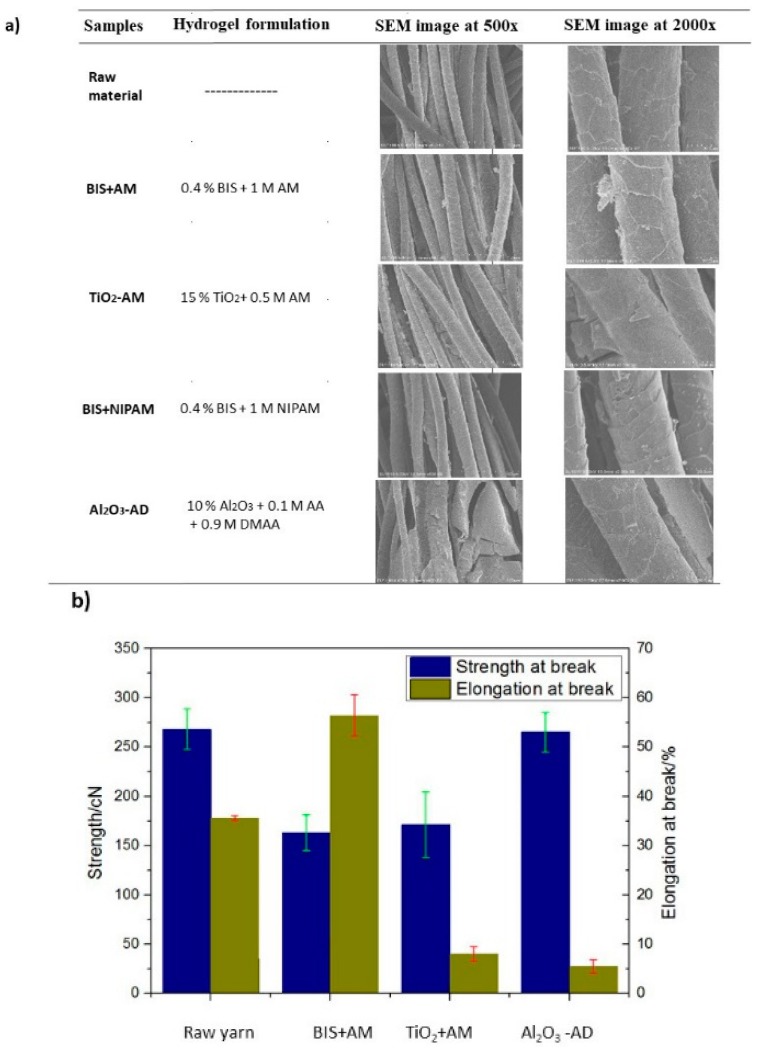
(**a**) SEM microphotographs at 500× and 2000× magnification of hydrogel-coated wool yarn using different hydrogel precursor formulations; (**b**) Strength and elongation of hydrogel-coated wool yarns.

Morphology of wool yarns coated by different hydrogel formulations was visualized by SEM images. From Figure 3a it is possible to verify that coating of the hydrogel onto wool yarns was very uniform, predicting it adhesion on the first layers of the wool fiber. In tensile tests (Figure 3b), the higher breaking elongation of BIS + AM hydrogel is greater than that TiO_2_-AM and Al_2_O_3_-AD hydrogels while the strength at break is greater with Al_2_O_3_-AD. This indicates that the hydrogel BIS + AM promotes a deformable yarn, so it is easy deformed. Usually, if strength increases, then elongation decreases and vice-versa. A higher strength means less deformable yarn, and breaks at low strain which is the case of hydrogel formulation Al_2_O_3_-AD.

The hydrogel formulation containing Al_2_O_3_-AD was investigated in the frame of this work since the strength of hydrogel-coated wool yarn was favored when two monomers were used (AA + DMAA) with the nano-alumina particles as crosslinking agent. Additionally, was analyzed the effect of different pre-treatments before the hydrogel coating on wool yarns.

The length variation of the hydrogel-coated wool yarns at wet and dry state was studied based on different pre-treatment conditions to achieve the best formulation under humidity conditions, for further characterization.

**Figure 4:**
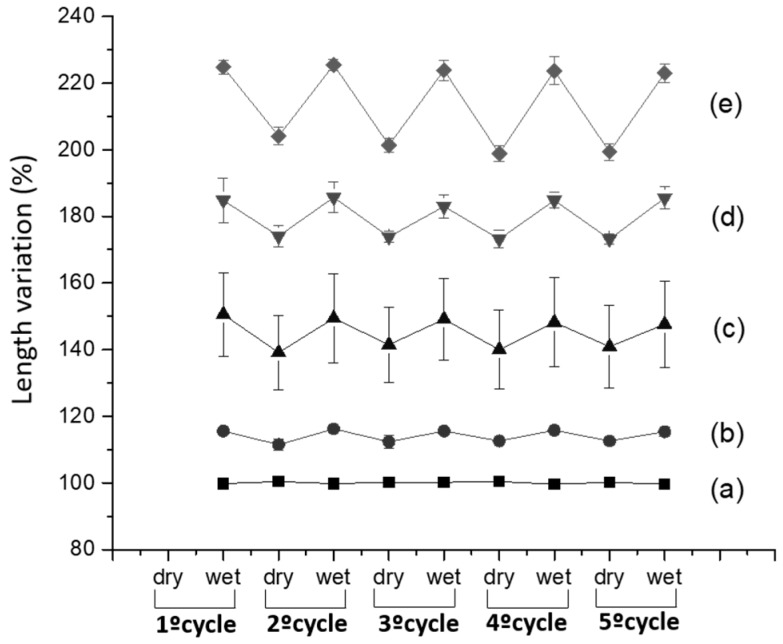
Length variation of hydrogel-coated wool yarns at wet and dry state during five consecutives cycles. Wool yarns were pre-treated with different concentrations of urea and sodium hydroxide: (**a**) 8 M urea, (**b**) 8 M urea with 0.0025 M NaOH, (**c**) 8 M urea with 0.005 M NaOH, (**d**) 8 M urea with 0.025 M NaOH, and (**e**) 8 M urea with 0.05 M NaOH during 2 h; after pre-treatment of yarns, UV light was used to trigger the polymerization on the surface of wool yarns forming the hydrogel. Formula of the hydrogel: 10% nano-Al_2_O_3_, 1% AA, 9% DMAA, 0.1% photo-initiator.

It was found that increasing the concentration of sodium hydroxide in the presence of urea lead to the increase of the length variation of wool yarns, for five cycles of dry-wet cycles. Polar solvents such as urea and alkaline pH solutions are well known because of their swelling and penetration promoting properties. They disturb inter- and intra-molecular hydrogen bonds, weak the hydrophobic interaction between polypeptides, thus leading to an exposure of more polypeptides chains of keratin to the solvent [21,22]. From Figure 4 it is perceptible that the length variation of the yarn increases as the increasing of concentration of the NaOH.

The production of the hydrogel network was formed by free-radical polymerization of multifunctional vinyl monomers (AA + DMAA). Each of these monomers contains a carbon double bond through which an active center may propagate to produce polymer chains. The method to generate active centers depends on the specific monomers, solvents and the reaction conditions employed [23]. Herein, it was based on UV light using photo-initiator Irgacure 2959. Photoinitiator has an essential role in the photopolymerization process, since it is excited under UV radiation which leads to the formation of the free radicals in the initiation step of the polymerization. The photo-initiator, Irgacure 2959, has been identified as extremely reactive, once been irradiated, it generates benzoyl radicals (higher reactivity than alkyl radicals) to initiate the polymerization, and also present high thermal stability [20]. It has also been suitable in achieving high cell viability during the photopolymerization reaction of cell-encapsulating hydrogels [17]. The hydrogel adhesion of the hydrogel coating remains stable up to 2 months.

Nano-alumina particles were used as crosslinking agent avoiding further use of cross-linkers in the polymerization reaction. Magnetically linked hydrogels, in which magnetic nanoparticles are used as crosslinking reagents, have been reported [15]. Metal oxide nanoparticles including alumina (Al_2_O_3_) can provide magnetic properties which can be suitable for biomedical applications [24]. The magnetic nanoparticles form covalent bonds with the polymer network due to the intrinsic strong negative charge of the acrylic acid [25]. This magnetic hydrogel network is suitable for humidity-induced dimensional changes of wool yarns, allowing the modification of elastic properties and changes in the shape of the sample [26].

**Figure 5:**
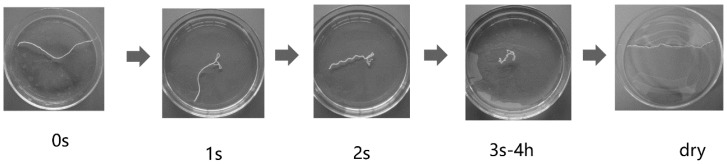
Images of sequential addition of water on hydrogel-coated wool yarns.

**Figure 6:**
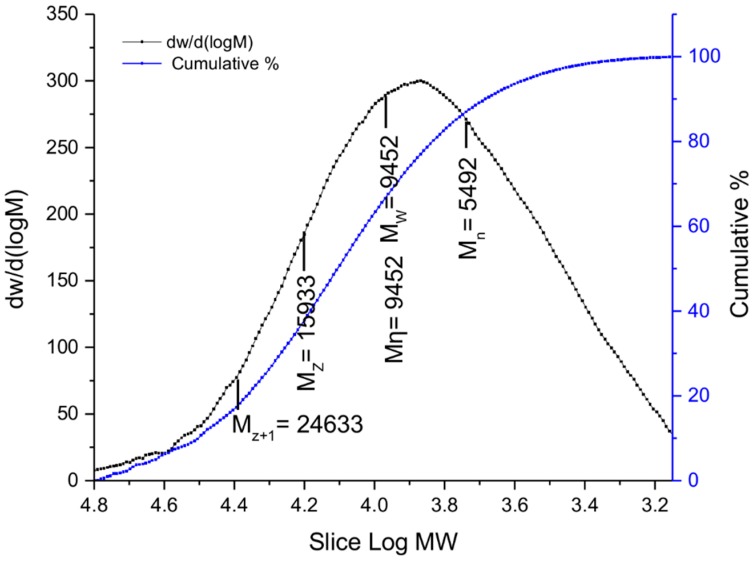
Molecular weight distribution curve for hydrogel (Nano-Al_2_O_3_-AD, 50 μL injected). Black line: differential curve; blue line: integral curve.

The dimensions of the hydrogel-coated wool yarns were changed after addition of water. These dimensional changes are easily perceptible from Figure 5. After a few seconds the wool yarns quickly curled after the addition of water and when dried elongate again. The molecular distribution of the hydrogel formulation was obtained by GPC/SEC. Figure 6 shows the molecular weight distribution curve for the hydrogel containing Nano-Al_2_O_3_-AD. The black line trace plots the molecular weight data as a differential curve, and the blue line trace plots that data as an integral curve. The weight-average molecular weight (Mw) and number-average molecular weight (Mn) were 9452 and 5492, respectively. In this case, the molecular weight distribution (polydispersity: *M*_w_/*M*_n_) was about 1.72, which indicates a moderate molecular weight distribution.

**Figure 7:**
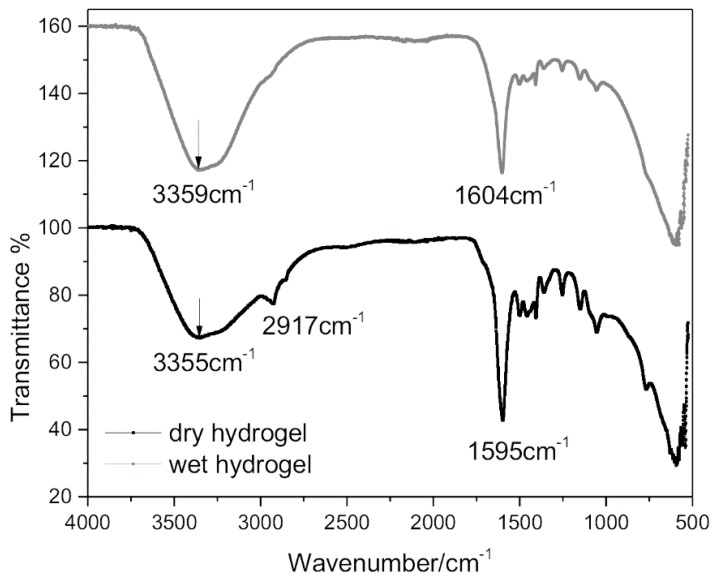
FT-IR spectrum of Nano-Al_2_O_3_-AD hydrogel in wet and dry state.

The hydrogel was characterized by FT-IR spectroscopy as showed in Figure 7. The presence of functional groups in the hydrogel had a crucial effect on its water-holding capacity. Consistent with the literature discussed, the disappearance of the characteristic vinyl bands at 1680 cm^−1^ in spectra indicates that all monomeric groups were involved in the polymerization reaction [27,28]. The intense peak at 3359 cm^−1^ and 3355 cm^−1^ (for wet and dry hydrogel, respectively) is the characteristic region of the O–H stretching vibration (region around at 3400–3300 cm^−1^). The peaks at 1604 cm^−1^ and 1595 cm^−1^ can be attributed to the adsorption of acrylic acid onto alumina, which is dominate by carboxylate group bridging to Al atoms [29]. Spectral features at 1590 cm^−1^ arise from the antisymmetric COO^−^ stretching vibrations of the carboxylate moiety of the acrylic acid.

**Figure 8:**
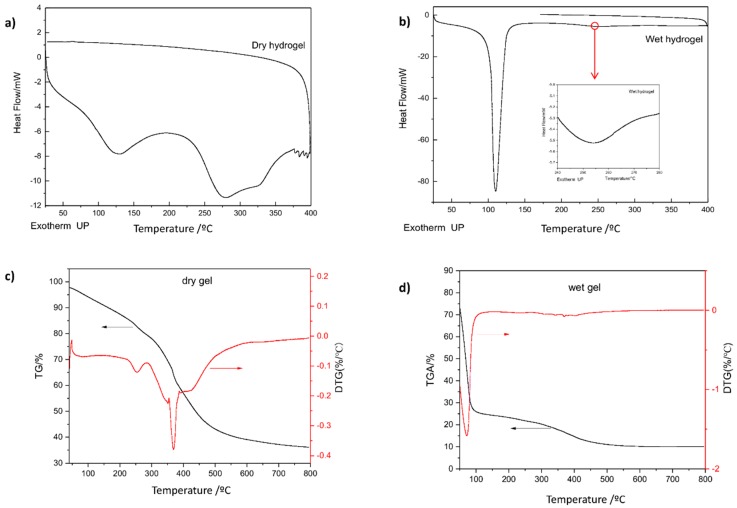
DSC curves of hydrogel based on two monomers: AA (acrylic acid) and DMAA (*N*,*N*-dimethylacrylamide), at wet and dry state: (**a**,**b**) DSC for dry and wet hydrogel, respectively; (**c**,**d**) TGA for dry and wet hydrogel, respectively.

For the thermal analysis measurement, DSC, the temperature range is from room temperature to 400 °C with a heating rate of 10 °C/min. These measurements were carried out under nitrogen atmosphere with flow rate of 30 mL/min. The weight of the samples varied from 5–10 mg and equilibrated at 25 °C. Figure 8 shows the DSC, TGA and DTG curves for hydrogel in dry and wet state. The DSC curves for dry hydrogel (Figure 8a) show an endothermic peak at about 120 °C and then an exothermic event (between 110 and 264 °C) related to the release of energy caused by mass loss. From Figure 8b (wet hydrogel) the DSC curve shows one sharp endothermic peak at about 110 °C and a slightly peak at around 250 °C. From Figure 8c dry hydrogel presents four events of degradation. The first one occurs between 55 to 110 °C with 5% water and moisture in the hydrogel; the second occurs between 110 to 250 °C with around 12% of mass loss; the third event occurs in the range of 250–450 °C with 38% of mass loss; and the fourth event from 450 to 800 °C occurs 10% of mass loss. According to Figure 8d, hydrogel in wet state presents three events of degradation. The first one is related to water loss (about 40%); the second event has a range of temperature (110–360 °C) related to a mass loss of about 10%; and the third event about 5% of mass loss.

Thus, it is possible to observe that wet hydrogel is more stable than dry hydrogel since remains steady with a large scale of temperature.

## 4. Conclusions

Coating of wool yarns by a polymeric hydrogel based on acrylic acid and *N*,*N*-dimethylacrylamide was successfully achieved by photopolymerization reaction. The reaction was conducted with nanoparticles of alumina as crosslinker and triggered by UV radiation. The success of crosslinking reaction was confirmed by FTIR analysis and the suitable polymer molecular weight distribution was established by GPC analysis. The length of hydrogel-coated wool yarns varied in the presence of water and, once dried, elongate again. These results revealed that hydrogel coating changes the dimensions of wool yarns under humidity conditions.

## Figures and Tables

**Table 1:** Length, elongation and strength variation of wool yarns samples coated on different hydrogel formulations. The percentage calculated is based on the original length of the yarn (50 mm). Five samples were tested for each sample.

Conditions		Formulation 1 (7% Acrylic Acid + 1% Alumina)	Formulation 2 (7% Acrylamide + 1% Titania)	Raw Yarns (Untreated)
**After polymerization**	Length (%)	98.5 ± 1.9	98.4 ± 2.1	100
	Elongation (mm)	33.2 ± 3.54	34.5 ± 2.58	35.4 ± 5.23
	Strength (cN)	372 ± 7.5	377 ± 8.2	365 ± 8.1
**Dry**	Length (%)	97.5 ± 3.1	96.9 ± 1.8	98.7 ± 1.5
	Elongation (mm)	30.1 ± 2.58	31.5 ± 1.76	34.5 ± 6.14
	Strength (cN)	356 ± 7.3	360 ± 10.3	365 ± 8.1
**95% Humidity**	Length (%)	99.2 ± 1.8	98.9 ± 1.3	100.5 ± 0.8
	Elongation (mm)	38.2 ± 4.56	36.2 ± 7.3	35.8 ± 6.12
	Strength (cN)	375 ± 8.5	364.2 ± 9.7	367 ± 5.2

**Table 2:** Length variation of hydrogel-coated wool yarns, after pre-treatment with urea (8 M) followed by hydrogel coating on the surface of wool yarns using UV irradiation to trigger the polymerization. Hydrogel precursor formulations: BIS-AM (0.4% BIS + 1 M AM); BIS-NIPAM (0.4% BIS + 1M NIPAM); Nano-TiO_2_-AM (15% TiO_2_ + 0.5 M AM) and Nano-Al_2_O_3_–AD (10% Al_2_O_3_ + 1% AA + 9% DMAA).

**(A) Under Different Humidity Conditions.**											
**Sample Name**	**Dry Yarn**	**25 °C 50% Humidity**		**25 °C 70% Humidity**		**25 °C 90% Humidity**		**25 °C 100% Humidity**		**Total Wet**	
Raw yarn	100%	100.2% ± 2.6%		100.0% ± 1.9%		100.1% ± 1.4%		99.9% ± 0.8%		99.8% ± 0.8%	
BIS-AM	100%	100.5% ± 0.9%		100% ± 1.4%		100.0% ± 0.7%		100.2% ± 1.7%		99.4% ± 1.5%	
BIS-NIPAM	100%	100.8% ± 1.6%		100.5% ± 1.8%		100.6% ± 1.7%		100.5% ± 0.9%		99.2% ± 1.4%	
TiO_2_-AM	100%	101.0% ± 1.8%		100.8% ± 2.5%		100.5% ± 1.5%		100.3% ± 2.7%		97.8% ± 1.3%	
Al_2_O_3_-AD	100%	100.8% ± 1.3%		101.0% ± 1.6%		100.5% ± 0.9%		100.2% ± 1.9%		94.2% ± 0.8%	
**(B) Under Five Cycles of Wet-Dry State.**											
**Sample Name**	**Dry Yarn (%)**	**1° Wet (%)**	**1° Dry (%)**	**2° Wet (%)**	**2° Dry (%)**	**3° Wet (%)**	**3° Dry (%)**	**4° Wet (%)**	**4° Dry (%)**	**5° Wet (%)**	**5° Dry (%)**
Raw yarn	100	99.8 ± 2.5	99.9 ± 2.1	99.9 ± 2.9	99.7 ± 3	100.0 ± 1.8	99.8 ± 1.9	100.1 ± 2.6	99.8 ± 2.4	100.2 ± 2.7	99.7 ± 0.9
BIS-AM	100	99.4 ± 1.8	99.2 ± 1.9	99.6 ± 2.4	99.5 ± 2.1	99.8 ± 2.5	99.7 ± 2.4	99.9 ± 1.8	99.7 ± 0.7	99.7 ± 0.7	99.6 ± 1.2
BIS-NIPAM	100	99.2 ± 1.9	99.0 ± 0.5	99.3 ± 1.8	99.0 ± 2	99.2 ± 0.6	99.1 ± 2.2	99.3 ± 1.6	99.5 ± 2.4	99.4 ± 1.4	99.2 ± 0.6
TiO_2_-AM	100	97.8 ± 0.7	96.5 ± 0.3	98.2 ± 1.0	97.9 ± 1.3	98.2 ± 1.6	97.8 ± 1.3	98.5± 1.9	98.1 ± 1.7	98.7 ± 1.7	98.5 ± 1.2
Al_2_O_3_-AD	100	94.2 ± 0.5	93.5 ± 1.6	95.6 ± 0.7	95.2 ± 1	96.4 ± 0.9	96.3 ± 0.8	97.5 ± 0.9	97.5 ± 0.9	98.3 ± 1	98.0 ± 1.5
